# Application and evaluation of a quality indicator system for pharmaceutical care in orthopedic perioperative pain management

**DOI:** 10.1371/journal.pone.0360057

**Published:** 2026-10-09

**Authors:** Qian Zhu, Panfeng Feng, Xia Chen, Jiaojiao Huang, Yong Chen

**Affiliations:** Department of Pharmacy, Affiliated Nantong Clinical College of Nantong University, Nantong First People’s Hospital, Nantong, Jiangsu, China; Shanghai Jiaotong University: Shanghai Jiao Tong University, CHINA

## Abstract

**Purpose:**

This study aimed to implement a previously developed quality indicator system for pharmaceutical care in orthopedic perioperative pain management and evaluate its impact on adherence to pharmaceutical care standards.

**Methods:**

A single-center before-and-after study with a historical control was conducted in 200 patients scheduled for elective orthopedic surgery. Pharmaceutical care quality was assessed using an established indicator system—developed in prior methodological work via the Delphi method and Analytic Hierarchy Process—in combination with the Technique for Order Preference by Similarity to Ideal Solution (TOPSIS). A retrospective pre-intervention cohort (n = 100, Nov 2024–Jan 2025) was evaluated to establish a reference, followed by targeted interventions. A prospective post-intervention cohort (n = 100, Apr–Jun 2025) was then evaluated. Groups were compared on overall adherence (*C*_*i*_ ≥ 0.6) to pharmaceutical care standards and full-score attainment rates of quality indicators. A sensitivity analysis was performed to assess the impact of documentation bias. Bonferroni correction was applied for multiple comparisons.

**Results:**

Overall adherence increased from 41% to 91% (risk difference, 50 percentage points; 95% CI, 37.83–60.05; *P* < 0.001), with a median difference of 0.32 (95% CI, 0.26–0.38). Sensitivity analysis confirmed the robustness of this finding: even under the most optimistic assumption for missing pre-intervention data, the post-intervention group remained higher (55% vs. 91%, *P* < 0.001). Four structural indicators showed descriptive improvements, with absolute increases ranging from 34 to 60 percentage points. Of the remaining 16 indicators, multimodal analgesia showed no significant difference (96% vs. 99%, *P* > 0.05). Among the other 15 process and outcome indicators, 13 remained significant after Bonferroni correction (*α* = 0.0031), with risk differences ranging from 14 to 33 percentage points. Two outcome indicators—achievement of target resting pain control and breakthrough pain incidence and management—did not reach the adjusted threshold, with improvements of 17 and 10 percentage points, respectively.

**Conclusion:**

Implementation of a structured quality indicator system was associated with meaningful improvements in pharmaceutical care adherence in orthopedic perioperative pain management. Sensitivity analysis supported the robustness of the finding. Improvements were observed across structural (descriptive), process, and outcome indicators, with the majority of process and outcome indicators showing significant gains after conservative correction. Study limitations include the historical control design, reliance on a composite measure, and single-center setting. These findings provide empirical support and practical reference for implementing structured quality assessment to improve pharmaceutical care adherence.

## Introduction

Pain management remains a significant challenge in orthopedic perioperative care. As one of the most common and distressing symptoms, pain is a critical determinant of postoperative recovery. Uncontrolled pain can trigger pathological remodeling of the central nervous system, increase myocardial oxygen consumption, and impair patients’ ability to eat, sleep, and mobilize, thereby delaying the recovery of cardiopulmonary function. Inadequate pain control not only hinders functional rehabilitation but also elevates the risk of secondary complications and the transition to chronic postoperative pain [1,2].

In recent years, a multidisciplinary pain management model involving anesthesiologists, surgeons, nurses, and clinical pharmacists has been widely adopted [3]. As members of this team, clinical pharmacists possess specialized pharmaceutical expertise, offering distinct advantages in medication recommendation, patient education, and treatment regimen optimization [4]. Evidence indicates that the participation of clinical pharmacists can improve perioperative pain management outcomes [5].

Nevertheless, numerous pharmaceutical-related challenges persist in clinical practice, including suboptimal utilization of patient-controlled analgesia (PCA), limited diversity in analgesic administration strategies [6], inappropriate use of analgesic agents [7], and a high incidence of adverse drug reactions (ADRs), such as postoperative nausea and vomiting and dizziness [8]. Furthermore, the integration of clinical pharmacists into perioperative pain management is relatively recent, and their roles are often hindered by deficiencies in operational models and implementation practices [4,9].

To address these challenges and better leverage the professional capabilities of clinical pharmacists, we previously developed a quality indicator system for pharmaceutical care in orthopedic perioperative pain management. This system was constructed using the Delphi method to consult multidisciplinary experts, and indicator weights were determined via the Analytic Hierarchy Process (AHP). This methodological work has been published in a Chinese-language journal [10].

The present study aims to implement this existing quality indicator system in clinical practice and evaluate its impact on adherence to pharmaceutical care standards. Specifically, we applied the system in combination with the Technique for Order Preference by Similarity to Ideal Solution (TOPSIS) to assess pharmaceutical care quality before and after targeted interventions. The TOPSIS method is a widely adopted approach for multi-criteria comprehensive evaluation. It integrates multiple indicators with varying weights by normalizing the raw data matrix and computing the relative proximity of each evaluation object to both the optimal and worst solutions [11–13]. Recognized for its ability to synthesize diverse measures into a single, interpretable score, this technique has been extensively applied in healthcare domains such as medical quality assessment, rational drug use, and performance appraisal [11,13–16]. In this study, TOPSIS was employed to enable comprehensive comparison of overall pharmaceutical care quality between patient groups before and after intervention.

By applying this previously developed quality indicator system in a before-and-after study with a historical control, we aimed to evaluate the impact of structured quality assessment and targeted interventions on adherence to pharmaceutical care standards. This implementation study provides empirical evidence on the practical utility of the indicator system and offers a reference for other institutions considering similar quality improvement initiatives.

## Patients and methods

### Study design and setting

This was a historically controlled, before-and-after study conducted at Nantong First People’s Hospital. A total of 200 patients undergoing elective orthopedic surgery under general anesthesia were included.

### Participants and eligibility criteria

The pre-intervention group comprised 100 patients whose data were retrospectively reviewed from November 2024 to January 2025. Following baseline evaluation of pharmaceutical care quality, targeted interventions were designed and implemented. The post-intervention group consisted of a separate cohort of 100 patients prospectively enrolled between April and June 2025.

Inclusion criteria were: age 18–75 years; American Society of Anesthesiologists (ASA) physical status classification I or II; and ability to comprehend and cooperate with pain assessment and education. Exclusion criteria included: history of psychiatric disorders or cognitive impairment; chronic pain conditions or substance abuse/dependence; and known allergy to any component of the prescribed analgesic medications.

### Sample size

The sample size was estimated based on the primary outcome (proportion of patients achieving *C*_*i*_ ≥ 0.6, as defined below). Similar studies evaluating quality improvement interventions in perioperative pain management reported effect sizes ranging from 21% to 35% [17–19]. Based on this evidence, and considering that our composite score (*C*_*i*_) may have a lower baseline rate than individual quality indicators, we conservatively assumed a baseline adherence rate of 45% and anticipated a 21-percentage-point improvement (to 66%). With 80% power and a two-sided alpha of 0.05, a minimum of 84 patients per group was required using a chi-square test for two proportions. Accounting for a potential 15% rate of missing data, the target sample size was approximately 99 patients per group. The final sample size was therefore set at 100 patients per group.

### Data collection

Data for the pre-intervention group were retrospectively extracted from electronic medical records, with data access commencing on 25/02/2025. For the post-intervention group, data were prospectively collected using an identical data collection form between 01/04/2025 and 30/06/2025. Data extraction was performed by two independent research assistants. Discrepancies were resolved through consensus with a third researcher.

### Ethics approval, registration, and informed consent

The study protocol was approved by the Medical Ethics Committee of Nantong First People’s Hospital (approval no. 2025XM074). The study was also registered in the National Medical Research Registration and Filing Information System (registration no. MR-32-26-022050). The requirement for written informed consent was waived for the pre-intervention group due to the retrospective nature of data analysis. Written informed consent was obtained from all participants in the prospectively enrolled post-intervention group. All data presented in this manuscript and any supplementary files have been anonymized to remove any personally identifiable information.

### Design of the perioperative pain management pharmaceutical care quality data collection form

Design a data collection form to record patient information: (1) Baseline Characteristics: name, hospital identification number, age, sex, weight, ASA physical status classification, surgical procedure, duration of surgery, and educational level; (2) Pain Education Status; (3) Pain Assessment Status; (4) Analgesic Medication Usage; (5) Pain Control Status; (6) Adverse Drug Reaction (ADR) Status. Any missing information was explicitly documented as “not recorded” to distinguish it from negative findings (e.g., “not done”).

### Application of the TOPSIS method for quality evaluation

Data were managed in Microsoft Excel. Based on the evaluation criteria for perioperative pain management pharmaceutical care quality (Table 2) and established literature [11,13,14], each indicator was assigned a score according to its evaluation result: For binary indicators: “Meets” = 10 points; “Does not meet” = 0 points. For three-level indicators: “Fully meets” = 10 points; “Partially meets” = 3, 5, or 7 points (with specific values determined by predefined rules based on the number and nature of criteria satisfied); “Does not meet” = 0 points. For indicators with conditionally applicable criteria, the scoring is adapted to the individual patient: the non-applicable criterion is treated as satisfied, and the patient is scored based on the remaining applicable criteria. See S2 Table for detailed scoring rules.

The TOPSIS analysis was performed following the procedures described in previous studies [12–14]. The raw scores were normalized using vector normalization according to Equation (1) to eliminate dimensional differences, where *X*_*ij*_ and *Z*_*ij*_ are the raw and normalized scores, respectively, of the *j*-th indicator for the *i*-th patient, with *i* = 1, 2,..., 100 and *j* = 1, 2,..., 20. Let *Z*_*j*_^+^ and *Z*_*j*_^-^ represent the maximum and minimum values of the *j*-th indicator across all patients, respectively, corresponding to the optimal and worst solutions under ideal conditions. *W*_*j*_ denotes the weight of the *j*-th indicator, derived from the AHP in our prior methodological work [10] and presented in Table 2. The weighted Euclidean distances from each patient’s indicator vector to the optimal solution (*D*_*ᵢ*_^+^) and to the worst solution (*D*_*ᵢ*_^-^) were calculated according to Equations (2) and (3), respectively. The relative proximity index *C*_*i*_ for each patient to the optimal solution was then calculated using Equation (4). The *C*_*i*_ value ranges from 0 to 1, with a higher value indicating better pharmaceutical care quality.


$$\begin{array}{c} Z_{ij\;}=\frac{X_{ij\;}}{\sqrt{\sum_{j=\text{1}}^{n}{X_{ij\;}}^{\text{2}}}} \end{array}$$ (1)

$$\begin{array}{c} D_{i}^{+}=\sqrt{\sum_{j=\text{1}}^{n}{W_{j}(Z_{j}^{+}-Z_{ij})}^{\text{2}}} \end{array}$$ (2)

$$\begin{array}{c} D_{i}^{-}=\sqrt{\sum_{j=\text{1}}^{n}{W_{j}(Z_{ij}-Z_{j}^{-})}^{\text{2}}} \end{array}$$ (3)

$$\begin{array}{c} C_{i}=\frac{D_{i}^{-}}{D_{i}^{+}+D_{i}^{-}} \end{array}$$ (4)

The overall quality was categorized into three levels for descriptive reporting [11,13,14]: High Adherent (*C*_*i*_ ≥ 0.8), Moderate Adherent (0.6 ≤ *C*_*i*_ < 0.8), or Non-Adherent (*C*_*i*_ < 0.6). For the primary outcome, patients with *C*_*i*_ ≥ 0.6 (combining High Adherent and Moderate Adherent) were considered Adherent to pharmaceutical care standards.

### Implementation of intervention measures

Following the initial evaluation of pharmaceutical care quality in the pre-intervention group, the following targeted interventions were implemented before the enrollment of the post-intervention cohort: (1) Establishment of a Multidisciplinary Pain Management Team: A clearly defined team comprising anesthesiologists, surgeons, nurses, and clinical pharmacists was formed. This team was responsible for developing a standardized perioperative pain management protocol for orthopedic surgery and conducting regular quality control assessments, with findings incorporated into staff performance evaluations. (2) Structured Professional Training and Assessment: Healthcare staff underwent periodic, specialized training. Knowledge retention and competency were assessed through written examinations. (3) Standardized Patient Education and Confirmation of Understanding: Patients and/or their families received and had pain management education materials explained to them. Healthcare personnel verified patients’ ability to perform pain self-assessment and operate PCA pumps correctly. (4) Protocolized Pain Assessment: Resting and dynamic pain assessments were conducted at required time points, documenting pain scores, location, and characteristics. (5) Integration of Medication Risk Assessment into the Admission Process: A standardized medication risk assessment was incorporated into the initial patient admission workflow. Physicians were required to formulate individualized analgesic regimens and ADR prevention plans based on this assessment. (6) Comprehensive Clinical Pharmacy Services: Clinical pharmacists delivered integrated care through active participation in ward rounds, providing patient medication education and monitoring, and conducting prescription review and evaluation. They were responsible for timely identification, intervention, and feedback on medication-related issues.

### Statistical analysis

Statistical analyses were performed using R version 4.3.0 (R Foundation for Statistical Computing, Vienna, Austria). All tests were two-sided, and a *P* value < 0.05 was considered statistically significant unless otherwise specified. Continuous variables were assessed for normality using the Shapiro-Wilk test; normally distributed variables are presented as mean ± standard deviation and compared using the independent samples *t*-test, while non-normally distributed variables are presented as median (Q1, Q3) and compared using the Mann-Whitney *U* test. Categorical variables are presented as frequencies (percentages). Nominal variables were compared using the Chi-square test (or Fisher’s exact test when expected cell counts were < 5), and ordinal variables were compared using the Mann-Whitney *U* test.

Baseline comparability was assessed using standardized differences (SMD), with SMD < 0.10 indicating good balance. For continuous variables, the Hodges–Lehmann estimator was used to estimate median differences with 95% confidence intervals. For binary outcomes, risk differences (RD) and their 95% confidence intervals were calculated using the Newcombe-Wilson method, with corresponding *P* values also reported. Four structural indicators reflecting institutional-level changes were presented descriptively without formal hypothesis testing. For the remaining 16 indicators subjected to statistical testing, Bonferroni correction was applied to control for multiple comparisons (*α* = 0.05/16 = 0.0031).

For indicators with three or more ordered score levels, the Cochran-Armitage test for trend was used to assess shifts in score distribution; exact *P* values were obtained via permutation tests (10,000 replications) when expected cell counts were < 5 in >20% of cells or total sample size < 40. Trend direction and magnitude were reported as *Z*-statistics. For these trend analyses, Bonferroni correction was applied for the 13 comparisons (adjusted *α* = 0.05/13 = 0.0038).

To assess robustness against documentation bias in the retrospective pre-intervention data, a sensitivity analysis was performed under a best-case scenario treating all missing data as fully compliant with the scoring rules.

## Results

### Baseline characteristics

The 200 patients in this before-and-after study comprised two cohorts: a pre-intervention group (n = 100, November 2024–January 2025) and a post-intervention group (n = 100, April–June 2025). Baseline characteristics were well balanced (Table 1; detailed data in S1 Table), with all SMD values < 0.10 except for surgery duration (SMD = 0.11, acceptable for non-randomized comparisons), and all *P* values > 0.05.

**Table 1:** Baseline characteristics of the study patients.

Characteristic	Pre-intervention Group (n = 100)	Post-intervention Group (n = 100)	SMD
Age, years, M (Q1, Q3)	61.00 (47.75, 67.00)	59.00 (51.50, 68.00)	0.07
Male, n (%)	46 (46.00)	43 (43.00)	0.06
Weight, kg, M (Q1, Q3)	66.00 (60.00, 75.00)	65.50 (60.00, 70.25)	0.04
ASA Physical Status, n (%)			
ASA I	6 (6.00)	5 (5.00)	0.04
ASA II	94 (94.00)	95 (95.00)	0.04
Anticipated Surgical Pain Intensity, n (%)			
Mild	36 (36.00)	32 (32.00)	0.08
Moderate	47 (47.00)	49 (49.00)	0.04
Severe	17 (17.00)	19 (19.00)	0.05
Surgery Duration, min, M (Q1, Q3)	90.00 (58.75, 116.25)	100.00 (60.00, 142.50)	0.11
Educational Level, n (%)			
≤Elementary	16 (16.00)	18 (18.00)	0.05
Secondary	47 (47.00)	49 (49.00)	0.04
≥College	37 (37.00)	33 (33.00)	0.08

**Notes:** Categorical data are presented as n (%); continuous variables with non-normal distribution are presented as median (Q1, Q3). SMD < 0.10 indicates good balance. Surgery duration showed SMD = 0.11, marginally above the threshold but within the acceptable range for non-randomized comparisons (SMD < 0.20).

**Abbreviation:** ASA, American Society of Anesthesiologists; SMD, standardized difference.

### Establishment of quality evaluation criteria for perioperative pain management pharmaceutical care

The established indicator system, developed via the Delphi method and AHP, consisted of three primary, seven secondary, and twenty tertiary indicators [10]. Based on clinical practice and practical considerations, we refined these findings into standardized perioperative pharmaceutical care quality criteria, with evaluation criteria presented in Table 2. The 20 indicators were classified as binary, three-level, or conditionally applicable types, with detailed scoring rules provided in S2 Table.

**Table 2:** Quality evaluation criteria for perioperative pain management pharmaceutical care.

Indicator Level 1 (Weight)	Indicator Level 2 (Combined Weight)	Indicator Level 3 (Combined Weight)	Evaluation Criteria	Evaluation Result
Structural Indicators (0.3000)	Staffing (0.1176)	Multidisciplinary Pain Management Team (0.0656)	① The patient receives multidisciplinary collaborative perioperative pain management involving anesthesiologists, surgeons, nurses, and clinical pharmacists.	☐ Meets ☐ Does not meet
		Clinical Pharmacist Coverage and Involvement (0.0519)	① Clinical pharmacists participate in the patient’s clinical management of perioperative pain. Routine review or evaluation alone is not clinical management.	☐ Meets ☐ Does not meet
	Policies and Training (0.1824)	Perioperative Pain Management Protocol/Pathway (0.1107)	① The patient is managed in accordance with the standardized perioperative pain management protocol/pathway.	☐ Fully meets ☐ Partially meets ☐ Does not meet
		Professional Education (0.0717)	① Healthcare personnel involved in the patient’s perioperative pain management have completed specialized training and competency assessments.	☐ Fully meets ☐ Partially meets ☐ Does not meet
Process Indicators (0.4000)	Pain Education (0.1215)	Patient Education on Perioperative Pain Knowledge and Assessment (0.0613)	① Education on perioperative pain knowledge and assessment methods is provided to the patient; ② The patient can correctly perform pain assessment using the NRS and FAS.	☐ Fully meets ☐ Partially meets ☐ Does not meet
		Patient Education on Analgesic Medications and Delivery Devices (0.0602)	① Education on analgesic medication is provided to the patient; ② The patient using PCA pump can correctly operate the device.	For patients using PCA pump: ☐ Fully meets ☐ Partially meets ☐ Does not meet For patients not using PCA pump: ☐ Meets ☐ Does not meet
	Pain Assessment (0.1181)	Resting Pain Assessment (0.0384)	① NRS scores are recorded upon admission, preoperatively, and daily postoperatively until discharge, along with descriptions of pain location and characteristics.	☐ Fully meets ☐ Partially meets ☐ Does not meet
		Dynamic Pain Assessment (0.0441)	① FAS scores are recorded upon admission, preoperatively, and daily postoperatively until discharge, along with descriptions of pain location and characteristics.	☐ Fully meets ☐ Partially meets ☐ Does not meet
		Pain Reassessment (0.0356)	① Pain scores are recorded during exacerbations and after analgesic adjustments, along with descriptions of pain location and characteristics.	☐ Fully meets ☐ Partially meets ☐ Does not meet
	Analgesic Medication (0.1604)	Multimodal Analgesia (0.0388)	① Implementation of multimodal analgesia using different types of analgesic drugs or techniques.	☐ Meets ☐ Does not meet
		Preventive Analgesia (0.0335)	① Administration of preventive analgesia throughout the perioperative period.	☐ Meets ☐ Does not meet
		Individualized Pharmacotherapy (0.0295)	① An analgesic regimen is individualized based on a risk assessment of analgesics considering the patient’s medical and medication history.	☐ Fully meets ☐ Partially meets ☐ Does not meet
		Prospective Prescription Review and Recommendations (0.0312)	① Perform a prescription review for analgesic use; ② Provide timely interventions and recommendations for any issues related to analgesic medications.	For patients with issues: ☐ Fully meets ☐ Partially meets ☐ Does not meet For patients without issues: ☐ Meets ☐ Does not meet
				Preventive and Corrective Measures for Adverse Drug Reactions (0.0274)	① Prophylactic management against analgesic-related ADRs is provided to the patient according to their individual risk status; ② Therapeutic management is promptly initiated for the patient experiencing analgesic-related ADRs.	For patients with ADR: ☐ Fully meets ☐ Partially meets ☐ Does not meet For patients without ADR: ☐ Meets ☐ Does not meet
Outcome Indicators (0.3000)	Analgesic Treatment Outcomes (0.1760)	Achievement of Target Resting Pain Control (0.0570)	① NRS scores are maintained at ≤3 consistently within 3 days post-surgery.	☐ Fully meets ☐ Partially meets ☐ Does not meet
		Achievement of Target Dynamic Pain Control (0.0608)	① FAS ratings are maintained at Grade A or B consistently within 3 days post-surgery.	☐ Fully meets ☐ Partially meets ☐ Does not meet
		Breakthrough Pain Incidence and Management (0.0582)	① Frequency of breakthrough pain episodes is ≤ 3 within 3 days post-surgery; ② Promptly implement management measures for the patient experiencing breakthrough pain.	For patients with breakthrough pain: ☐ Fully meets ☐ Partially meets ☐ Does not meet For patients without breakthrough pain: ☐ Meets
	Analgesic Medication Evaluation (0.1241)	Retrospective Prescription Evaluation and Feedback (0.0414)	① Conduct a prescription evaluation for analgesic use; ② Provide timely feedback to physicians on inappropriate analgesic use.	For patients with inappropriate use: ☐ Fully meets ☐ Partially meets ☐ Does not meet For patients without inappropriate use: ☐ Meets ☐ Does not meet
		Appropriateness of Analgesic Drug Use (0.0503)	① Appropriate Analgesic Selection: Combination therapy, ADR prophylaxis for moderate-to-high-risk patients, and individualized pharmacotherapy; ② Accurate Analgesic Administration: Single dose, frequency, route, and treatment duration; ③ Therapy Adjustment: Dosage or drug change based on evolving pain status.	☐ Meets ☐ Does not meet
		Monitoring and Evaluation of Adverse Drug Reactions (0.0323)	① Conduct ADR monitoring and evaluation for the patient.	☐ Fully meets ☐ Partially meets ☐ Does not meet

**Note:** Scoring: For binary indicators: “Meets” = 10 points; “Does not meet” = 0 points. For three-level indicators: “Fully meets” = 10 points; “Partially meets” = 3, 5, or 7 points (with specific values determined by predefined rules based on the number and nature of criteria satisfied); “Does not meet” = 0 points. Appropriateness of Analgesic Drug Use is binary: “Meets” requires all three criteria; any unmet yields “Does not meet”. For indicators with conditionally applicable criteria (patient education on analgesic medications and delivery devices, prospective prescription review and recommendations, preventive and corrective measures for adverse drug reactions, breakthrough pain incidence and management, and retrospective prescription evaluation and feedback), the scoring is adapted to the individual patient: the non-applicable criterion is treated as satisfied, and the patient is scored based on the remaining applicable criteria. See S2 Table for detailed scoring rules for all indicators.

**Abbreviations:** NRS, Numerical Rating Scale; FAS, Functional Assessment Scale; PCA, patient-controlled analgesia; ADRs, adverse drug reactions.; ADR, adverse drug reaction.

### Evaluation results of pharmaceutical care quality in perioperative pain management

The distribution of *C*_*i*_ values for perioperative pain management pharmaceutical care quality is presented in Table 3 (the raw scores of each indicator for each patient are provided in S3 Table). In the pre-intervention group, *C*_*i*_ values ranged from 0.2 to 1.0, with 41.00% (41/100) of patients achieving *C*_*i*_ ≥ 0.6 (defined as Adherent to pharmaceutical care standards), indicating suboptimal baseline quality. In the post-intervention group, *C*_*i*_ values ranged from 0.3 to 1.0, with 91.00% (91/100) attaining *C*_*i*_ ≥ 0.6. The difference between groups was significant (RD: 50.00 percentage points; 95% CI: 37.83–60.05; χ^2^ = 55.704, *P* < 0.001). The median *C*_*i*_ value increased from 0.57 (0.34, 0.69) in the pre-intervention group to 0.87 (0.78, 1.00) in the post-intervention group. The Mann-Whitney *U* test showed a statistically significant difference between groups (*U* = 8823, *Z* = −9.361, *P* < 0.001), with a Hodges-Lehmann median difference of 0.32 (95% CI: 0.26–0.38). These findings suggest a notable improvement in overall adherence following the intervention.

**Table 3:** Distribution of *C*_*i*_ values and adherence categories for pharmaceutical care quality in perioperative pain management.

*C*_*i*_	Pre-intervention group (n = 100)	Post-intervention group (n = 100)
**Distribution**		
1	2 (2.00)	29 (29.00)
0.9 ≤ *C*_*i*_ < 1.0	1 (1.00)	12 (12.00)
0.8 ≤ *C*_*i*_ < 0.9	4 (4.00)	23 (23.00)
0.7 ≤ *C*_*i*_ < 0.8	17 (17.00)	19 (19.00)
0.6 ≤ *C*_*i*_ < 0.7	17 (17.00)	8 (8.00)
*C*_*i*_ < 0.6	59 (59.00)	9 (9.00)
**Summary**		
High Adherent (*C*_*i*_ ≥ 0.8)	7 (7.00)	64 (64.00)
Moderate Adherent (0.6 ≤ *C*_*i*_ < 0.8)	34 (34.00)	27 (27.00)
Non-Adherent (*C*_*i*_ < 0.6)	59 (59.00)	9 (9.00)
**Statistics**		
Median (Q1, Q3)	0.57 (0.34, 0.69)	0.87 (0.78, 1.00)
Median difference (Post- Pre) (95% CI)	—	0.32 (0.26 − 0.38)

**Note:** Data are presented as n (%). *C*_*i*_ is the relative closeness to the ideal solution calculated by TOPSIS, ranging from 0 to 1, with higher values indicating better pharmaceutical care quality. Adherent (*C*_*i*_ ≥ 0.6) combines High Adherent (*C*_*i*_ ≥ 0.8) and Moderate Adherent (0.6 ≤ *C*_*i*_ < 0.8). Median (Q1, Q3) and median difference (95% CI) are presented as summary statistics for the *C*_*i*_ distribution. The median difference was estimated using the Hodges-Lehmann estimator.

To assess the potential impact of documentation bias, we performed a sensitivity analysis under the most optimistic assumption for missing pre-intervention data—treating all missing documentation as fully compliant with the scoring rules. Under this scenario, the proportion of patients with *C*_*i*_ ≥ 0.6 in the pre-intervention group increased from 41.00% (41/100) in the primary analysis to 55.00% (55/100). Even under this extreme assumption, the post-intervention group (91.00%, 91/100) remained higher (χ^*2*^ = 32.877, *P* < 0.001). This suggests that while documentation bias may have partially contributed to the observed improvement, it cannot fully explain its magnitude (see S4 Table).

### Achievement status of each indicator

For each quality indicator, we calculated the number of patients who achieved a score of 10 points, corresponding to “Meets” for binary indicators, “Fully meets” for three-level indicators, and the equivalent full-score designation for conditionally applicable indicators as defined in S2 Table.

For structural indicators—multidisciplinary pain management team, clinical pharmacist coverage and involvement, perioperative pain management protocol/pathway, and professional education—notable improvements were observed, with absolute increases ranging from 34.00 to 60.00 percentage points. Formal statistical testing was not performed for these indicators due to unit-of-analysis considerations; improvements are presented descriptively.

Excluding structural indicators, the remaining 16 indicators were analyzed. Among these, multimodal analgesia showed a ceiling effect and was not significant (96.00% vs. 99.00%; RD, 3.00 percentage points; 95% CI, −2.07 to 8.89; *P* = 0.369). The other 15 process and outcome indicators all showed significant improvements in uncorrected comparisons (all *P* < 0.05). After Bonferroni correction (*α* = 0.0031), 13 of the 15 indicators remained significant, with RD ranging from 14.00 to 33.00 percentage points and 95% confidence intervals excluding zero. Two outcome indicators—achievement of target resting pain control (*P* = 0.006) and breakthrough pain incidence and management (*P* = 0.048)—did not reach the adjusted threshold, despite absolute improvements of 17.00 and 10.00 percentage points, respectively (95% CI: 4.76–28.60 and 0.01–19.95). See Table 4 for detailed results.

**Table 4:** Comparison of full-score attainment rates of pharmaceutical care quality indicators between the pre- and post-intervention groups.

Quality Indicator	Pre-intervention group (n=100)	Post-intervention group (n=100)	Absolute Improvement	RD (95% CI)	χ^2^	*P*	Sig.
**Structural Indicators**							
Multidisciplinary Pain Management Team	29 (29.00)	89 (89.00)	60.00	—	—	—	—
Clinical Pharmacist Coverage and Involvement	56 (56.00)	90 (90.00)	34.00	—	—	—	—
Perioperative Pain Management Protocol/Pathway	20 (20.00)	66 (66.00)	46.00	—	—	—	—
Professional Education	9 (9.00)	48 (48.00)	39.00	—	—	—	—
**Process Indicators**							
Patient Education on Perioperative Pain Knowledge and Assessment	65 (65.00)	88 (88.00)	—	23.00 (11.36, 33.95)	14.713	<0.001	S
Patient Education on Analgesic Medications and Delivery Devices	54 (54.00)	86 (86.00)	—	32.00 (19.54, 43.17)	24.381	<0.001	S
Resting Pain Assessment	63 (63.00)	84 (84.00)	—	21.00 (8.81, 32.42)	11.321	<0.001	S
Dynamic Pain Assessment	54 (54.00)	80 (80.00)	—	26.00 (13.04, 37.80)	15.287	<0.001	S
Pain Reassessment	48 (48.00)	77 (77.00)	—	29.00 (15.67, 40.92)	17.941	<0.001	S
Multimodal Analgesia	96 (96.00)	99 (99.00)	—	3.00 (-2.07, 8.89)	—	0.369	NS
Preventive Analgesia	80 (80.00)	94 (94.00)	—	14.00 (4.71, 23.45)	8.665	0.003	S
Individualized Pharmacotherapy	57 (57.00)	81 (81.00)	—	24.00 (11.24, 35.74)	13.464	<0.001	S
Prospective Prescription Review and Recommendations	68 (68.00)	90 (90.00)	—	22.00 (10.83, 32.65)	14.587	<0.001	S
Preventive and Corrective Measures for Adverse Drug Reactions	55 (55.00)	80 (80.00)	—	25.00 (12.08, 36.81)	14.245	<0.001	S
**Outcome Indicators**							
Achievement of Target Resting Pain Control	65 (65.00)	82 (82.00)	—	17.00 (4.76, 28.60)	7.419	0.006	NS
Achievement of Target Dynamic Pain Control	58 (58.00)	79 (79.00)	—	21.00 (8.14, 32.94)	10.219	0.001	S
Breakthrough Pain Incidence and Management	80 (80.00)	90 (90.00)	—	10.00 (0.01, 19.95)	3.922	0.048	NS
Retrospective Prescription Evaluation and Feedback	77 (77.00)	98 (98.00)	—	21.00 (12.27, 30.27)	20.160	<0.001	S
Appropriateness of Analgesic Drug Use	75 (75.00)	95 (95.00)	—	20.00 (10.32, 29.73)	15.686	<0.001	S
Monitoring and Evaluation of Adverse Drug Reactions	55 (55.00)	88 (88.00)	—	33.00 (20.79, 43.96)	26.721	<0.001	S

**Note:** Data are presented as n (%). Absolute Improvement and RD are presented in percentage points. 95% confidence intervals for RD were calculated using the Newcombe-Wilson method. Sig. = significant after Bonferroni correction (*α* = 0.0031); S = significant, NS = not significant. Fisher’s exact test for Multimodal Analgesia. Structural indicators: no statistical testing due to unit-of-analysis; improvements shown descriptively. For conditionally applicable indicators (patient education on analgesic medications and delivery devices, prospective prescription review and recommendations, preventive and corrective measures for adverse drug reactions, breakthrough pain incidence and management, and retrospective prescription evaluation and feedback), the proportions reported in this table reflect the full-score attainment rates across both applicable and non-applicable subgroups combined. Detailed subgroup-specific data are presented in S5 Table.

Cochran-Armitage trend tests were performed to assess shifts in score distributions across ordered categories for indicators with three or more score levels. Of the 13 eligible indicators, 12 showed significant linear improvements (*P* < 0.05), with *Z*-statistics ranging from 2.657 to 5.442. After Bonferroni correction (*α* = 0.0038), 9 of the 13 indicators remained significant. The most pronounced improvements were observed for monitoring and evaluation of adverse drug reactions (*Z* = 5.442, *P* < 0.001), pain reassessment (*Z* = 4.370, *P* < 0.001), and dynamic pain assessment (*Z* = 3.878, *P* < 0.001). No significant trend was found for breakthrough pain incidence and management (*Z* = 1.391, *P* = 0.180). Full details are provided in S5 Table.

## Discussion

Perioperative pain management is a multidisciplinary process whose quality is influenced by a complex interplay of factors. While previous studies have established valuable insights into nursing practices in this area [18,20], the pharmaceutical care component has remained underexplored. Clinical pharmacists, with their specialized expertise, are uniquely positioned to address this gap through patient education, therapeutic optimization, and treatment monitoring [19,21]. However, the impact of these services depends largely on implementation quality. In this study, we applied a previously developed quality indicator system—constructed via Delphi and AHP—in combination with TOPSIS to evaluate pharmaceutical care in orthopedic perioperative pain management. The composite *C*_*i*_ score enabled overall comparison of care quality before and after intervention, while individual indicators (Table 4) provided detailed insights into specific strengths and weaknesses.

The pre-intervention evaluation revealed notable deficiencies in pharmaceutical care quality, with only 41.00% of patients achieving *C*_*i*_ ≥ 0.6. Detailed analysis identified multiple systemic issues that guided our targeted interventions: (1) absence of a formal multidisciplinary pain management team and a standardized perioperative pain management protocol or pathway; (2) insufficient specialized expertise among healthcare personnel in performing specific pain management duties; (3) overreliance on verbal patient education, resulting in 29 patients demonstrating poor comprehension of pain self-assessment or PCA pump operation; (4) incomplete pain assessment documentation (11 without pain scores, and 14 with pain scores but lacking pain location or characteristics); (5) inadequate medication risk management (18 lacking risk assessments and preventive measures); (6) deficiencies in pharmaceutical care oversight (15 inappropriate prescriptions lacking prospective review and timely intervention, and 20 inappropriate prescriptions lacking retrospective evaluation and feedback); (7) suboptimal analgesic use (5 inappropriate selections, 20 improper dosing); and (8) inadequate ADR monitoring (27 lacking documented monitoring and evaluation). These findings are consistent with reports from other Chinese healthcare settings [6–9,22,23] and provided a clear roadmap for intervention design.

After the intervention, the proportion of patients achieving *C*_*i*_ ≥ 0.6 increased from 41.00% to 91.00% (RD, 50.00 percentage points; 95% CI, 37.83–60.05; *P* < 0.001), with a median difference of 0.32 (95% CI: 0.26–0.38). Sensitivity analysis supported this finding: even when all missing pre-intervention data were treated as fully compliant, the post-intervention group remained higher (55.00% vs. 91.00%, *P* < 0.001). These results suggest that the intervention was associated with a notable improvement in overall adherence to pharmaceutical care standards.

At the individual indicator level, full-score attainment rates for the 20 indicators were examined to identify specific areas of improvement. Four structural indicators—implemented at the departmental level—were reported descriptively, with absolute increases ranging from 34.00 to 60.00 percentage points. Of the remaining 16 indicators, multimodal analgesia showed no significant difference between groups (96.00% vs. 99.00%, *P* = 0.369). The other 15 process and outcome indicators all showed improvements before correction for multiple comparisons (all *P* < 0.05); after Bonferroni correction (*α* = 0.0031), 13 remained significant, with RD ranging from 14.00 to 33.00 percentage points. These results suggest that improvements occurred across multiple domains. Most of these reflect the implementation of care processes—including improvements in pain education, assessment, pharmacotherapy, prescription management, and safety monitoring. Notably, the outcome indicator achievement of target dynamic pain control also improved from 58.00% to 79.00% (RD, 21.00 percentage points; 95% CI, 8.14–32.94; *P* = 0.001), suggesting potential clinical relevance, as it directly reflects patients’ pain control.

Multimodal analgesia showed high attainment in both groups (96.00% vs. 99.00%), suggesting a ceiling effect that limits its discriminatory power. Its inclusion nonetheless serves a supportive role, suggesting that this standard of care has been maintained in routine practice. Future iterations could refine this indicator to capture implementation quality—such as appropriateness of drug combinations or timing [24]—rather than mere presence.

Two outcome indicators—achievement of target resting pain control (*P* = 0.006) and breakthrough pain incidence and management (*P* = 0.048)—did not reach the Bonferroni-adjusted threshold (*α* = 0.0031). Their absolute improvements were 17.00 and 10.00 percentage points, respectively. The first indicator narrowly missed the corrected threshold, likely reflecting the conservative nature of the Bonferroni correction. For breakthrough pain incidence and management, the relatively low event rate (80.00% vs. 90.00% attainment) may have limited statistical power [25], contributing to its borderline uncorrected significance. These findings highlight the value of considering both statistical significance and clinical relevance when interpreting results from multiple comparisons.

Beyond binary attainment rates, Cochran-Armitage trend tests further showed that 12 of 13 eligible indicators had significant linear improvements (all *P* < 0.05); after Bonferroni correction (*α* = 0.0038), 9 remained significant, suggesting systematic shifts toward higher score categories across most indicators.

Despite overall improvement, certain indicators remained below 80% attainment after the intervention, including professional education, perioperative pain management protocol/pathway, pain reassessment, and achievement of target dynamic pain control. These areas represent priorities for sustained quality improvement, which could be addressed through intensified professional training, integration of protocols into clinical pathways, and more frequent quality audits [26,27].

Several limitations should be considered. First, the before-and-after design with a historical control group limits causal inference, as temporal factors or unmeasured organizational changes may have contributed to the observed results; although no other pain management initiatives were formally implemented during the study period, we cannot entirely exclude such influences. Additionally, since staff were aware of being observed only during the post-intervention phase, Hawthorne effects cannot be excluded [28]. Second, the indicator system has not been externally validated, having been applied in a single institution, and the indicators and weights were derived from expert elicitation, which may limit generalizability. For structural indicators, although assessed at the patient level, patients within each cohort shared the same institutional context, which may affect the precision of statistical estimates; we therefore did not perform formal hypothesis testing for these indicators and report their improvements descriptively. Third, the primary outcome—the TOPSIS-derived *C*_*i*_ score—is a composite measure of adherence to pharmaceutical care standards, rather than a direct measure of clinical outcomes such as opioid consumption, adverse drug events, mobilization, hospital length of stay, readmission, or persistent postoperative pain. Moreover, most of the individual indicators reflect processes and practices that were directly targeted by the intervention. Thus, while we observed notable improvements in these quality indicators, we cannot conclude that these necessarily translate into tangible clinical benefits. Additionally, while TOPSIS provides a useful composite measure, it may obscure specific deficiencies by aggregating indicators—a concern also raised regarding composite endpoints in clinical trials, where combining multiple outcomes into a single measure may mask the effects on individual components [29]. To address this, we reported individual indicator achievement rates alongside the composite score. Fourth, the intervention consisted of multiple components implemented simultaneously—including multidisciplinary team establishment, staff training, patient education, standardized pain assessment, medication risk assessment, clinical pharmacist involvement, and quality audit procedures—making it impossible to isolate the contribution of any single component, including whether the observed changes reflect the indicator system or pharmacist involvement. Finally, retrospective data collection may have introduced documentation bias; however, sensitivity analysis suggests this alone does not explain the observed improvement.

Despite these limitations, this implementation study suggests that a structured quality indicator system can be associated with meaningful improvements in adherence to pharmaceutical care standards in orthopedic perioperative pain management. For other institutions adopting this framework, we suggest: (1) adapting indicators, cut-offs, and weights to local contexts; (2) refining indicators with ceiling effects to capture implementation quality rather than mere presence; (3) ensuring independent data collection to minimize bias; and (4) conducting periodic reviews to retire outdated indicators and introduce new ones. Further research is warranted to assess generalizability across diverse settings and to disentangle the effects of individual intervention components.

## Conclusion

Implementation of a structured quality indicator system was associated with meaningful improvement in adherence to pharmaceutical care standards in orthopedic perioperative pain management. The improvements were supported by sensitivity analysis. Improvements were observed across structural (descriptive), process, and outcome indicators, with the majority of process and outcome indicators showing significant gains after conservative correction. Study limitations include the historical control design, reliance on a composite measure, and single-center setting. Future research could validate the indicator system against hard clinical outcomes using multicenter designs and further investigate which components are most critical for driving change. Despite these limitations, our findings provide empirical support for using structured quality assessment to improve adherence to pharmaceutical care standards and offer a practical reference for institutions considering similar quality improvement initiatives.

## Associated data

This section collects any data citations, data availability statements, or supplementary materials included in this article.

## Supporting information

S1 Table Detailed baseline characteristics of the study patients. (XLSX)

S2 Table Detailed scoring rules for binary, three-level, and conditionally applicable quality indicators. (XLSX)

S3 Table Raw scores of the 20 quality indicators for each patient in the pre- and post-intervention groups. (XLSX)

S4 Table Sensitivity analysis of *C*_*i*_ distribution and adherence categories under optimistic assumptions for missing pre-intervention data (missing treated as fully compliant). (XLSX)

S5 Table Complete distribution of three-level and conditionally applicable indicators across score categories. (XLSX)
